# Pain score distribution: an expert elicitation study for distal humerus hemiarthroplasty and total elbow arthroplasty using a validated numerical patient rated outcome-measure in trauma

**DOI:** 10.1016/j.jseint.2026.101640

**Published:** 2026-01-27

**Authors:** Adam Watts, Zaid Hamoodi, Catriona McDaid, Catherine Hewitt, PEDANT Expert Panel, Lars Adolfsson, Jonathan Evans, Toni Luokkala, Pierre Mansat, Joideep Phadnis, Alexander van Tongel, Andrew Wright

**Affiliations:** a Faculty of Health and Social Care Medicine, Edge Hill University, Ormskirk, England; b Upper Limb Unit, Wrightington Wigan and Leigh Teaching Hospital NHS Foundation Trust, West Lancashire, England; c Department of Health Sciences, University of York, York, England; d York Trials Unit, University of York, York, England

**Keywords:** Total elbow arthroplasty, Pain score, Distal humerus hemiarthroplasty, Elbow, Acute trauma, Expert elicitation

## Abstract

**Hypothesis:**

With equal numbers of adults with unreconstructable distal humerus fractures undergoing total elbow arthroplasty (TEA) and distal humerus hemiarthroplasty (DHH) a randomized trial is needed to compare the interventions. Patients have identified pain as the key outcome. There are no data on pain outcomes using a validated pain instrument to plan a trial. This study aims to use expert elicitation to produce probability distributions for the Patient Rated Elbow Evaluation (PREE) pain score at 12 months in adults undergoing TEA or DHH for trauma.

**Methods:**

Using the Sheffield Expert Elicitation Framework seven experts were recruited and provided with a summary of current knowledge on pain outcomes from a systematic review. They were asked to consider their median of the PREE pain score at 12 months after a TEA for acute trauma, their difference in the median PREE pain scores between TEA and DHH, and their standard deviation (SD) of the PREE pain score after TEA and DHH. The rational independent observer concept was used to achieve consensus.

**Results:**

The elicited median value for the PREE pain at 12 months after TEA for acute trauma was 13.6/50 (interquartile range 10.6 to 16.8). The estimated median of the difference in PREE pain scores was 3.4 (interquartile range −0.3 to 7.1). The elicited standard distribution of PREE pain scores was 9 (SD 2.2), and after DHH was 12 (SD 3.0).

**Conclusion:**

Probability distributions for pain outcomes measured using the PREE pain subscale following DHH and TEA for acute trauma in adults generated by expert elicitation indicate an expectation of small differences between interventions. A noninferiority randomized trial using the PREE pain subscale as the primary outcome at 12 months could be conducted to explore whether the possible advantages of DHH, such as lower cost and reduced adverse events, are supported.

Fractures of the distal humerus are increasing due to an ageing population, and elbow arthroplasty for trauma has doubled in the ten years that the National Joint Registry has been collecting data for England, Wales, Northern Ireland, and Guernsey with 144 confirmed elbow arthroplasty operations for acute trauma in 2022.^1^^,^^22^ The treatment options for this injury are cast immobilization without an operation, fixation with plates and screws (osteosynthesis), and elbow prosthetic arthroplasty.^27^ Where the bone cannot be fixed there are two options for elbow arthroplasty for trauma, distal humerus hemiarthroplasty (DHH) and total elbow arthroplasty (TEA). TEA has been the standard care but there are concerns about limitations in loading the operated joint, the risk of the implant loosening from the bone over time, polyethylene wear, implant, or periprosthetic fracture, leading to pain and a need for revision surgery.^5^ DHH has been proposed as an alternative solution with the hypothesis that the risks caused by joint loading activities and of implant loosening and revision is reduced because only 1 part of the joint being replaced.^23^ Hemiarthroplasty has been used in other joints for trauma, but has been shown to be associated with higher risk of joint pain and inferior functional outcomes when compared to total joint arthroplasty, although modes of failure may be different from the elbow.^25^^,^^32^ There are no large randomized controlled trials comparing the outcome of DHH to TEA in acute elbow trauma and it is not known which approach is better for patients.

One of the barriers to conducting a randomized trial is the relatively low incidence of distal humerus fractures requiring arthroplasty, which makes it challenging to recruit and randomize sufficient participants to adequately power the study in an economically viable time period. An alternative is to use Bayesian methodology, whereby we start with an initial probability distribution for the outcome (prior distribution), collect data (likelihood) and apply Bayes theorem to update the probability distribution (posterior distribution).^10^ The posterior distribution should be closer to the true population distribution than the prior, based on the evidence collected. When using this approach, we can use an uninformative prior, where every outcome has the same probability, which is akin to the frequentist approach, where we ignore any previous knowledge on the subject in the analysis other than for conducting power calculations. In most cases, however, we have some knowledge of the expected outcomes, and it may be possible to produce an informative prior based on published data and expert knowledge, whereby some outcomes are considered to have a higher probability than others.

Trial planning requires identification of a primary outcome which should be valid, feasible to measure, and relevant to the participants and end-users.^17^ In the CODER Real-Time Delphi consensus study patients identified pain as the primary outcome domain for elbow arthroplasty.^31^ Of the validated pain instruments for elbow arthroplasty, the Patient Rated Elbow Evaluation (PREE) best fulfils the criteria for the primary outcome instrument.^16^ The PREE measures the average amount of pain over the last week using an 11-point numerical rating scale (NRS) from no pain (0) to worst pain ever (10) in five domains; worst pain, rest pain, pain when lifting a heavy object, pain with repeated movement, and frequency of pain from never (0) to always (10). However, a lack of data on PREE scores in the population undergoing elbow arthroplasty for acute trauma is a barrier to trial planning.

A systematic review of the published literature since 2000 has shown that the estimate of pain outcomes after elbow arthroplasty for trauma is not well-described.^30^ The literature consists largely of case series with implicit high risk of bias. The identified studies use either an NRS or Likert scale for pain. None report the pain scores using the PREE. Expert elicitation is a scientific model used to produce probability distributions for unknown parameters. A number of different approaches can be used such as the Classic Method and the Sheffield Expert Elicitation Framework (SHELF).^8^^,^^11^^,^^18^ In the elicitation process experts are asked to consider what is currently known on a subject, and their own experience as an expert, in order to elicit a probability distribution for quantities of interest for which the value is not known.^19^ This elicited information can be used to inform prior distributions for Bayesian approaches or to inform sample size calculations with a frequentist approach.^21^^,^^28^

The aim of this study is to undertake expert elicitation using the SHELF methodology to produce prior probability distributions for the PREE pain score at 12 months in adult patients with unreconstructible distal humerus fractures treated with TEA or DHH.

## Methods

The protocol adheres to the reference protocol for expert elicitation studies described by Bojke et al in 2021 which was funded by the National Institute for Health Research Health Technology Assessment programme.^6^

### Experts

The experts were recruited from specialists in orthopedic elbow surgery. The experts were chosen on the basis of known expertise in elbow arthroplasty for distal humeral fracture with evidence of research on this topic and from specialist shoulder and elbow surgeons with a strong background in research methods evidenced by publication. Representation was balanced between those predominantly using DHH and those predominantly using TEA for acute trauma reconstruction. Experts were drawn from a number of different countries to ensure a breadth of opinion was sampled. Experts were required to be able to communicate in English and understand written English.

Letters of invitation were sent via email outlining the protocol and time commitment required. Experts had to be willing to participate in one-to-one sessions and commit to attending a group elicitation session. The experts were asked to declare any personal or financial conflicts of interest.

### Approach to elicitation

The SHELF was used.^20^ The experts were provided with a standardized SHELF expert briefing document that explained the process of expert elicitation and introduced the key concepts that the expert needed to understand for the workshop (Supplementary Appendix S1). Additional training was available online via the SHELF website which participants were signposted to (https://shelf.sites.sheffield.ac.uk/e-learning-course).

The experts were provided with an evidence dossier developed by AW based on a systematic review summarizing current evidence regarding pain outcomes for TEA and DHH in trauma from studies published since 2000 (Supplementary Appendix 2). The evidence consists largely of observational studies of case series, and pain was measured entirely with either 11-point NRS pain or 4- or 5-point Likert scales. The summary evidence from the systematic review reporting a high risk of bias was provided for the experts, showing summary mean NRS pain scores and 95% confidence intervals for TEA and DHH, as well as summary Likert pain scale outcomes reporting the proportion reporting pain as none, mild, moderate, and severe.

To help the experts convert the summary evidence using the Likert scale into PREE outcomes, data from a separate study by Angst et al of a nontrauma population were presented that had measured pain six months after TEA using PREE pain and a Likert pain scale from the 36-Item Short Form Health Survey (SF-36).^4^ The mean PREE pain score was 14/50 (standard deviation [SD] 13.3) and the mean SF-36 pain score was 60 which equates to “mild pain” on the 6-item SF-36 Likert scale. The experts were invited to comment on the data presented and suggest changes prior to starting the elicitation workshops to produce a final evidence dossier that was shared with all reviewers.

One-to-one elicitation was conducted by A.W. as facilitator and Z.H. as recorder with the experts to elicit the quantities of interest.

Once all the experts had completed their one-to-one elicitation exercises, they were brought together online for a group elicitation meeting to discuss the anonymized individual results and to elicit a distribution for each quantity of interest as a group using the principle of the “rational independent observer” (RIO).

### Elicitation method

Variable interval methods were used to elicit probability distributions for the median NRS pain scores for DHH and TEA.^6^ The median value was elicited, rather than the mean, as it is easier for experts to consider the value below or above which they would expect 50% of the values to lie. In normally distributed data the median will approximate to the mean. The quartile method was used for initial elicitation by individual experts. The probability method was used for the group elicitation. SDs were elicited using the method described by Alhussain and Oakley.^2^

### Quantities of interest

A: Experts were asked to consider their median of the PREE pain score at 12 months after a TEA.

B: Experts were asked to consider their difference in the median PREE pain scores at 12 months in patients having TEA or DHH for acute trauma.

C: Experts were asked to consider their SD of the PREE pain score at 12 months after TEA and DHH.

### Fitting

The fitting was guided by the expert's beliefs using SHELF software and distribution parameters generated, the beta distribution was chosen as the default because this produces distribution bounded by the lower and upper plausible limits provided by the expert. The individual fitted distribution was shown to the expert without commentary or revision. For the group stage the quantity of interest was elicited using the RIO model and the fitted consensus RIO distribution was shared with the experts for consideration and adjustment if it did not adequately reflect their consensus view. A beta distribution was generated from the parameters elicited from the experts, bounded by the parameter limits.

### Aggregation

Mathematical aggregation was not undertaken as there was insufficient agreement between the individually elicited fitted distributions. Behavioral aggregation was used with experts attending an online consensus meeting to agree a fitted distribution for the RIO.

### Documentation

Standardized SHELF documents were used to collect data from the experts before and during the elicitation process. The “Expert Enquiry” form was completed by the experts prior to the elicitation meeting and their responses collated (Supplementary Appendix S3). This included the expert's name and title, their background and expertise, and their declarations of interest. The experts were asked to review the evidence dossier and to list any additional sources of evidence that they felt should be considered by the experts, and to list any questions, clarifications, or corrections pertaining to the evidence dossier.

During each workshop the SHELF elicitation record documents were used. The purpose of the elicitation was clearly defined and a record of the pre-elicitation training received by each expert was documented, including any training elicitations. The expertise of all participants was recorded alongside declarations of interest. The evidence document was reviewed and clarifications made without discussion of the quality or interpretation of the evidence. The structure of the elicitation was discussed with the expert, with clear definitions of the quantities of interest provided.

### One-to-one elicitation

The SHELF elicitation record was completed for each quantity of interest. This captured the date, start time, and finish time of the meeting, a record of the discussion and parameters elicited. The facilitator (A.W.), an elbow surgeon trained in SHELF methodology, led the discussion. To ensure anonymity of the experts' elicitations at the group feedback stage, each was assigned a unique identifier that was used in feedback.

For each quantity of interest, the definition and the evidence regarding only that quantity was reviewed. The expert was asked to record their lower and upper plausible limits for that quantity. The expert was then asked to consider and record their median (M) and lower and upper quartiles (Q1, Q2) for the quantity of interest using preprepared slide sets from the SHELF package to explain these concepts. The recorder (Z.H.) documented the experts' responses and then fitted a distribution using SHELF software to the expert's assessment which was captured in the form of density functions. The expert was shown the fitted distribution but not invited to revise it and no commentary was provided by the facilitator at this stage.

SDs for the PREE pain at 12 months for TEA and DHH were elicited by asking the expert to consider their lower and upper estimates of the proportion of patients scoring between their elicited median score for PREE pain after TEA and their median plus 10 points. For the SD of DHH their median PREE pain score was calculated from the sum of their median for TEA and their difference in PREE pain score between TEA and DHH at 12 months. The expert was asked to consider their lower and upper estimates of the proportion of patients scoring between their elicited median score for PREE pain after DHH and their median plus 10 points. The assurance shiny application from the SHELF website, a previously validated tool for model assurance, was used to calculate their upper and lower limit of the SDs from the proportions estimated.^2^

### Group elicitation

The group elicitation record from SHELF was used to document the group elicitation exercise. The facilitator computed an equally-weighted average of the density functions (linear pool) using SHELF software. This was not revealed to the experts, but available as a benchmark by the facilitator if group discussions lead to an unwarranted narrowing of the uncertainty that was not supported by the group elicitations. The groups were shown each of the individually synthesized fitted distributions for each quantity of interest in an anonymized format to stimulate discussion. The experts were given the opportunity to give their opinion about the quality and interpretation of the evidence.

For the group stage elicitation, the limits were set to the parameter limits for the quantity of interest; the median PREE at 12 months after TEA (0 to 50) and the RIO difference in PREE scores at 12 months between TEA and DHH (−50 to 50). The group elicitation method and judgements were recorded. The group consensus judgements for RIO using the probability method were recorded for the probabilities (P1, P2, P0) of three values of the quantity of interest (X) chosen by the facilitator (X1, X2, X0).

To elicit the SD for PREE pain scores for TEA and DHH at 12 months the group were asked to reach a consensus on what a RIO would consider to be a plausible upper and lower limit for the SD of PREE pain scores at 12 months after TEA and DHH, based on the experts lower and upper limits from the individual elicitation stage, which was recorded as the “group plausible range”. The quartile method was used to elicit the RIO probability distribution of the quantities of interest.

The parameters for the fitted distribution for the consensus RIO judgement was recorded in the SHELF documentation, and the fitted distribution shared with the experts alongside some implied probabilities such as the 10th and 90th percentile. The experts were given the opportunity to adjust the elicited parameters with further rounds of fitting and feedback if required until the final fitted distribution was accepted and recorded in the chosen distribution field.

### Delivery

The structured elicitation exercises and group elicitation stage was conducted online using Zoom (Zoom Video Communications Inc.) to accommodate expert surgeons from Belgium, France, Finland, Sweden, and the United Kingdom.

### Training and piloting

Online training videos for self-directed learning were made available (https://shelf.sites.sheffield.ac.uk/e-learning-course) and experts were encouraged to review these before the one-to-one elicitation meetings. To familiarize the expert to the methods of expert elicitation a trial elicitation was conducted to elicit parameters for a quantity of interest unrelated to the study aims at the start of the one-to-one elicitation workshop. This allowed the facilitator to highlight common errors of elicitation such as overconfidence (bounds between upper and lower plausible limits too narrow) and imprecision (eg, placing upper and lower quartiles too far from the median).

The protocol was piloted with a local elbow surgeon expert (A Wright) to ensure that the procedures function as intended and could be easily followed by the participants.

## Results

Seven experts were contacted and agreed to take part in the expert elicitation. Prior to participation in the elicitation workshop all experts read the SHELF elicitation guide and one expert accessed the online SHELF training material. The experts declared any conflicts of interest (Supplementary Appendix S3). All experts were happy with evidence dossier provided and none provided any additions, corrections, or sought any clarifications.

### Median PREE pain score at 12 months following TEA for distal humerus fracture

The experts were asked to consider their distribution of the median score on the PREE pain scale from 0 to 50 (were 0 represents no pain) at 12 months after TEA. They were asked to consider what the distribution would look like if 100 experiments were conducted and all the medians were plotted. The parameters elicited from the experts are given in Table I.

**Table I:** Individual expert elicited median Patient Rated Elbow Evaluation scores at 12 months following total elbow arthroplasty for acute distal humerus fracture.

Expert	Lower plausible limit	Lower quartile	Median	Upper quartile	Upper plausible limit	Fitted beta distribution parameters		
						α	β	μ
A	10	12	15	16	18	0.953	0.825	14.40
B	10	12	16	20	26	0.711	1.070	15.70
C	5	8	10	12	16	1.670	1.960	9.97
D	10	12	15	17	20	0.908	1.000	14.60
E	7	12	14	16	26	3.650	6.090	14.00
F	10	13	16	18	22	1.310	1.410	15.70
G	12	14	15	16	18	2.160	2.160	15.00

At the group stage this elicited data were considered and there was discussion of the available evidence. The parameter limits for the PREE are 0 and 50. The experts were asked to agree RIO cumulative probabilities for a value of the median PREE of 10, 16, and 20 and agreed values of 0.2, 0.7, and 0.9. The fitted beta distribution curve (parameters a = 6.69, ß = 17.4) had a median value of 13.6/50 (interquartile range 10.6 to 16.8). This was fed back to the experts, with the implications that 10% of the median values would lie below 7.9 and 10% would lie above 19.6, and the experts felt that this reasonably represented the RIO distribution. No further elicitation was conducted for this quantity.

### Difference in the median PREE pain score at 12 months between TEA and DHH for distal humerus fracture

The experts were asked to consider their distribution of the difference in the median PREE pain scores in 100 trials comparing TEA and DHH at 12 months. The parameters elicited are provided in Table II.

**Table II:** Individual expert elicited difference in the median Patient Rated Elbow Evaluation score at 12 months between total elbow arthroplasty and distal humerus hemiarthroplasty for acute distal humerus fracture.

Expert	Lower plausible limit	Lower quartile	Median	Upper quartile	Upper plausible limit	Fitted beta distribution parameters		
						α	β	μ
A	1	2	3	4	5	1.000	1.000	3.00
B	0	2	5	8	12	0.824	1.080	4.86
C	−5	2	5	7	10	2.480	1.450	4.82
D	5	7	10	15	20	0.655	0.980	10.30
E	−5	−2	0	2	8	1.870	2.850	−0.06
F	0	3	5	7	10	1.530	1.530	5
G	−6	−2	0	2	6	2.160	2.160	0

The parameter limits for the median difference of the PREE pain scores were −50 and 50. The experts were asked to agree RIO cumulative probabilities for a value of the median PREE of 0, 4, and 10 and agreed values of 0.3, 0.5, and 0.925. The fitted beta distribution curve (parameters a = 44.5, ß = 38.9) had a median value of 3.4 (interquartile range −0.3 to 7.1). This was fed back to the experts, with the implications that 10% of the difference values would lie below −3.6 and 10% would lie above 10.4, and the experts felt that this reasonably represented the RIO distribution and no further elicitation was required.

### Distribution of the SDs of the PREE pain score at 12 months following TEA and DHH

To elicit the SDs the experts were asked to consider their estimate of the proportion of patients that would score between their median and their median plus 10 points. Table III shows the 5% and 95% probability estimates of the proportion scoring in this interval for each expert.

**Table III:** Individual expert elicited distribution of the standard deviations of the Patient Rated Elbow Evaluation pain score at 12 months following total elbow arthroplasty and distal humerus hemiarthroplasty.

Expert	Probability of scores falling between median, median+10 TEA		Elicited standard distribution PREE pain for TEA		Probability of scores falling between median, median+10 DHH		Elicited standard distribution PREE pain for DHH	
	5%	95%	5%	95%	5%	95%	5%	95%
A	0.30	0.45	11.9	6.1	0.25	0.40	14.8	7.8
B	0.20	0.40	19.1	7.8	0.20	0.40	19.1	7.8
C	0.20	0.45	19.0	6.1	0.10	0.40	39.5	7.8
D	0.25	0.45	14.8	6.1	0.25	0.45	14.8	6.1
E	0.40	0.48	7.8	4.9	0.35	0.45	9.7	6.1
F	0.30	0.499	11.9	4.3	0.15	0.499	26.0	4.3
G	0.40	0.45	7.8	6.1	0.35	0.40	9.7	7.8

*TEA*, total elbow arthroplasty; *DHH*, distal humerus hemiarthroplasty; *PREE*, Patient Rated Elbow Evaluation.

In the group stage the experts were asked to consider the proportions elicited and from these to elicit a RIO distribution using the quartile method. For TEA the experts agreed a lower limit for the standard distribution of 7 and an upper limit of 20. The RIO median was 12, lower quartile 10, and upper quartile 20. These parameters were fitted to a normal distribution eliciting a mean SD for PREE pain scores at 12 months after TEA of 9 (SD 2.2). For DHH the experts agreed a lower limit of 7 and upper limit of 20. The RIO median was 12, lower quartile 10, and upper quartile 14. These parameters were fitted to a normal distribution eliciting a mean SD for PREE pain scores at 12 months after DHH of 12 (SD 3.0). The experts were satisfied that the elicited distributions represented their views.

## Discussion

Elbow prosthetic arthroplasty is used for unreconstructible intra-articular distal humerus fractures in patients who are fit for surgical intervention. According to the National Joint Registry data, in the most recent three years reported, when patients undergo elbow arthroplasty for trauma, approximately half receive TEA and half DHH.^1^ There are some apparent differences in the demographics with 22% of those undergoing DHH under 65 years of age and 43% over 74 years, compared to 9% <65 years for TEA and 62% >74 years.^1^ The proportion of female patients is 84% for both interventions from the latest report.^1^ This data indicates some preference for use of DHH in younger patients, which is consistent with a concern regarding long term risks of ulna implant loosening that is present with TEA. There is a lack of quality evidence to compare TEA to DHH to help the clinician and patient choose the best intervention. One randomized trial with 20 patients in each treatment arm, has reported equivalent functional outcomes from TEA and DHH for acute fractures with no difference in pain outcomes, but the trial has been found to be at high risk of bias using the Cochrane ROB-2 criteria and may be underpowered to detect a difference.^15^^,^^26^ From this, some may conclude that it does not matter which intervention is used, but there are differences in the reported revision rate for TEA and DHH at 2 years in favor of TEA reported in the NJR, and systematic reviews have raised concerns regarding functional outcomes in younger patients, and the overall levels of pain after DHH.^1^^,^^12^^,^^30^ Others have reported that function scores may be higher after DHH compared to TEA.^7^ This expert elicitation study suggests that pain outcomes measured by the PREE pain scale at 12 months will exhibit small differences between the two interventions. There may be as yet undetermined benefits to DHH, for example the absence of an ulna component may reduce ulna related adverse events and overall costs. This uncertainty regarding the best treatment option for patients with unreconstructible intra-articular distal humerus fractures could be addressed by a suitably powered noninferiority prospective randomized controlled trial stratified by age, but this may not be feasible due to low incidence. Bayesian trial methodology may be a more feasible approach for this type of rare intervention.

A recent Delphi consensus study with patient and public involvement has reported that the most important outcome for patients after elbow arthroplasty is pain. This was felt to be more important than elbow function.^31^ The choice of instruments to measure pain after TEA is limited. Many instruments combine pain assessment with function assessment (Oxford Elbow Score, Disabilities of the Arm, Shoulder and Hand score, Liverpool elbow score) and pain items are rarely reported separately by investigators.^9^^,^^14^^,^^24^ Pain scales such as the visual analog scale, NRS, or Likert scale have been validated as tools for measuring pain, but we are not aware of any investigations of their psychometric properties in elbow arthroplasty studies and they are unidimensional.^13^ The PREE and American Shoulder and Elbow Surgeons scores measure and report pain independently, over several dimensions, and have been validated in general elbow conditions and in TEA studies.^3^^,^^4^^,^^29^ The American Shoulder and Elbow Surgeons does not capture frequency of pain symptoms and includes physician assessment of strength, range of movement and stability making it more cumbersome, therefore the PREE has been selected for use in this study.

Angst et al have reported that in patients undergoing TEA the mean PREE improved from a score of 30/50 (SD 9.2) pre-operation to 15/50 (SD 13.7) at 6 months after surgery. However, the study population was not representative of those undergoing arthroplasty for trauma with 54% of the 79 patients included having a diagnosis of rheumatoid arthritis.^3^ Acute fracture patients were excluded from the analysis and this data can only guide the expert as to the likely outcome in the trauma population.

Expert elicitation is a tool to produce probability distributions for unknown quantities of interest. The experts in the PEDANT study have provided their consensus based on a synthesis of current knowledge, their own expertise and the views of other experts in the field, to provide estimated median PREE pain scores for patients 12 months after TEA, the difference between median PREE pain scores for TEA and DHH and estimates of the SDs for both. The experts have expressed their view that a RIO, considering the evidence provided, observing their elicited quantity probability distributions and listening to the considerations made by the experts in the group discussion would conclude that in a randomized trial comparing pain outcomes at 12 months in patients undergoing TEA or DHH for acute distal humerus fractures the median pain score for TEA would be 14/50 with a variance of 81, for DHH the median score would be 17/50 with a variance of 144. Applying these parameters to the Bayesian assurance application developed by Alhussain and Oakley, it is unlikely that a randomized trial will show benefit of one intervention over the other if pain is the primary outcome.^2^ It is possible that the background data is misleading or that the parameters elicited by the experts underestimate the treatment effect. It is also important to note that behavioral aggregation was used to agree a fitted distribution for the RIO between the experts. Even if well-managed this type of aggregation may be a source of social and cognitive bias such as group polarization, overconfidence and groupthink and hence is a possible limitation of this study. Nevertheless, this data provides prior distributions that can be updated using Bayesian methodology in a future randomized controlled trial for this rare intervention, to provide more accurate estimates of population probability distributions.

## Conclusion

Probability distributions for pain outcomes measured using the PREE pain scale following DHH and TEA for acute trauma in adults generated by expert elicitation indicate an expectation of small differences between interventions. A noninferiority randomized trial using the PREE pain subscale as the primary outcome at 12 months could be conducted to explore whether the possible advantages of DHH, such as lower cost and reduced adverse events, are supported.

## Acknowledgments

PEDANT expert panel: Professor Lars Adolfsson, MD. Professor of Orthopaedics, University of Linkoping, Sweden. Mr. Jonathan Evans, PhD. Senior Clinical Lecturer, University of Exeter and Consultant Shoulder and Elbow Surgeon, Princess Elizabeth Orthopaedic Centre, UK. Dr. Toni Luokkala MD, PhD. Consultant Elbow and Hand Surgeon, Hospital Nova, Jyvaskyla, Finland. Professor Pierre Mansat MD PhD. Professor of Orthopaedics and Traumatology, University Hospital, Toulouse, France. Mr. Joideep Phadnis PhD. Consultant Elbow and Shoulder Surgeon, Unievrsity Hospitals Sussex, Brighton, UK. Honorary Reader Brighton and Sussex Medical School, Brighton, UK. Professor Dr. Alexander van Tongel, MD, PhD. Consultant Shoulder and Elbow Surgeon, University Hospital Ghent, Ghent, Belgium. Mr. Andrew Wright, MD. Consultant Shoulder and Elbow Surgeon, Wrightington Wigan and Leigh Teaching Hospitals NHS Foundation Trust, UK.

## Disclaimers

Funding: No outside funding or grants were received to assist in this study.

Conflicts of interest: Adam Watts receives payment from Stryker for educational activities. His institution has received payment for research unrelated to this manuscript. The authors, their immediate families, and any research foundations with which they are affiliated have not received any financial payments or other benefits from any commercial entity related to the subject of this article.

Given his role as Editor in Chief, Pierre Mansat had no involvement in the peer-review of this article and has no access to information regarding its peer-review. Full responsibility for the editorial process for this article was delegated to William Mallon.
