# Supplementary material for: Pain score distribution: an expert elicitation study for distal humerus hemiarthroplasty and total elbow arthroplasty using a validated numerical patient rated outcome-measure in trauma
Source: JSES Int. 2026 Jan 27;10(3):101640. doi: 10.1016/j.jseint.2026.101640 (PMC13054023; doi:10.1016/j.jseint.2026.101640)
Supplement: Supplementary Appendix S1 [file mmc1.docx]

# Appendix 1: SHELF Expert Briefing

Thank you for agreeing to take part in an expert knowledge elicitation workshop. You will be one of a group of experts who will be asked to make judgements regarding one or more Quantities of Interest (QoIs). The QoIs for your workshop, and the importance of expert knowledge about these quantities, have been set out in your invitation letter. The purpose of this document is to explain what kind of judgements you will be asked to make.

## SHELF

The elicitation will be conducted following the Sheffield Elicitation Framework (SHELF), based on elicitation methodologies originally recommended in O’Hagan et al (2006) and subsequently refined through extensive practical experience with the SHELF approach.

The SHELF process begins with inviting experts and agreeing with them a date for the elicitation workshop. In preparation for the workshop, experts are sent this briefing document and details of the uncertain quantities of interest that will be considered in the workshop. Another important step is the gathering of evidence; you may already have received a draft ‘evidence dossier’ and been asked if you can contribute additional evidence.

The workshop will be conducted by an experienced facilitator who will begin by reviewing the QoIs and the final dossier of evidence relating to them. For each QoI, the facilitator will then ask all the experts to make a number of judgements which will reveal the range of opinion regarding the quantity. The facilitator will then lead a discussion aimed at exploring and understanding differences, with a view to reaching a set of ‘consensus’ judgements to represent the combined knowledge of the experts present.

It is important to note that you will *not* be asked to provide single estimates of any of these quantities. The elicitation process will instead involve considerations such as what a plausible range of values would be for each quantity of interest, and which values, in your opinion, are more likely than others. You may have considerable uncertainty about some of these quantities (though less than that of a lay person). This will not be of concern during the elicitation itself, as the outputs from the elicitation will reflect large uncertainty when it is present.

To illustrate the kind of judgements you will be asked to make in the workshop we imagine a freshwater biologist who is participating as an expert in a workshop where one of the quantities of interest is the average lifetime of a particular species of fish in ideal water conditions (ideal temperature, oxygenation, acidity, etc.). We will call this quantity L.

## Probabilities

The formal way to describe and quantify uncertainty is by using probabilities, and we begin by considering how the biologist’s knowledge and uncertainty about L are described by probabilities.

The concept of probability is invariably introduced to students using the *frequency* definition, according to which the probability of something happening is the frequency with which is happens in the long run, over a very long sequence of repetitions. For instance, the probability of throwing a ‘Six’ with a regular six-sided die is one-sixth, because in the long run, over very many tosses of the die, ‘Six’ will come up on one throw in six.

The frequency definition of probability only applies to events, such as tossing a die, which are *repeatable* so that we can observe how often the event occurs. However, the quantities of interest that you will be asked about in the workshop will only ever have one value. The fish average lifetime L is a typical example. The average lifetime in ideal conditions is a quantity that can only have one value. (Lifetimes of individual fish obviously vary but the average has a unique fixed value.) We cannot define the probability of the fish living on average more than 3 years by the frequency with which the average is more than 3 years in a long sequence of repetitions because it is not repeatable. The average lifetime *either is or is not* more than 3 years.

Every QoI that we ask about in expert elicitation has a single fixed true value, although that value is unknown. Probabilities in elicitation are therefore not frequency probabilities but are instead judgements. If the biologist gives a probability, say 0.4 or 40%, that the average lifetime of this species of fish is more than 3 years, this is a judgement that the biologist makes, expressing his or her degree of certainty. A probability close to 1 will mean that the biologist is almost sure that L will exceed 3, while a probability near 0 will mean that he or she is almost sure that L will *not* exceed 3. A value of 0.4 would mean that this expert judges it to be slightly less likely that L will be more than 3 than that it will be less than 3.

Notice that different experts will generally have different beliefs and will therefore assign different probabilities to statements such as “L is more than 3”. In elicitation, we use a definition of probability called *personal probability* or *subjective probability*.

## Subjectivity

You may feel uncomfortable about this element of subjectivity in the elicitation process, because subjectivity has negative connotations in everyday usage, including bias, prejudice, wishful thinking or sloppy thinking. So it is important to understand how these risks are addressed in a well-conducted elicitation exercise.

- It is inevitably true that different experts will give different probabilities, because they evaluate the evidence differently and because they bring different experiences and expertise to bear on the QoI. Some experts’ judgements will be better than others. You have been invited to take part in this elicitation exercise because the organisers believe that your expertise is particularly valuable and that your judgements will be good.
- Judgements are usually elicited from several experts, thereby achieving greater objectivity by accessing a broader spectrum of opinion.
- The available evidence regarding the QoIs will be carefully reviewed, in order that each expert is fully aware of any relevant data.
- The SHELF elicitation process is designed to avoid the kinds of psychological bias that are known to be most common in eliciting probability judgements.
- The elicitation will be conducted by a facilitator who is skilled in managing the process to help experts to make the best possible judgements.

## Judgements

The facilitator will choose what judgements to ask from the experts, within the SHELF guidance. You may be asked to make direct judgements of probability, such as, “What is your probability that L is greater than 3?” However, you will generally also be asked more indirect questions.

The facilitator will often ask experts for their *plausible range*, comprising a lower plausible limit L and an upper plausible limit U. The idea is that you should be almost certain that the QoI will lie in this range.

Another judgement that the facilitator may request is your median. This is a value M such that you think the QoI is equally likely to be above or below M.

Whatever judgements the facilitator requests will be fully explained, with clear guidance on how to make those judgements accurately. You will also do a practice elicitation before having to make your judgements about the quantities of interest, in order to familiarise you with the task.

*We hope that you will find the elicitation exercise interesting. Thank you again for agreeing to take part!*

Reference:

O'Hagan, A., Buck, C. E., Daneshkhah, A., Eiser, J. E., Garthwaite, P. H., Jenkinson, D. J., Oakley, J. E. and Rakow, T. (2006). *Uncertain Judgements: Eliciting Expert Probabilities*. Chichester: Wiley.
