# Supplementary material for: Pain score distribution: an expert elicitation study for distal humerus hemiarthroplasty and total elbow arthroplasty using a validated numerical patient rated outcome-measure in trauma
Source: JSES Int. 2026 Jan 27;10(3):101640. doi: 10.1016/j.jseint.2026.101640 (PMC13054023; doi:10.1016/j.jseint.2026.101640)
Supplement: Supplementary Appendix S2 [file mmc2.docx]

**Appendix 2: Evidence dossier for Pain score distribution Expert elicitation study for Distal humerus hemiarthroplasty and total elbow Arthroplasty using a validated Numerical patient rated outcome-measure in Trauma (PEDANT)**

Adam C Watts

Catriona McDaid

Catherine Hewitt

Version 1.5, 7^th^ Jan 2024

**Quantities of interest**

A: You will be asked to consider your median of Patient Rated Elbow Evaluation (PREE) pain at 12 months after a total elbow replacement.

B: You are being asked to consider your difference in the median PREE pain scores at 12 months in patients having a total elbow replacement or distal humerus hemiarthroplasty for acute trauma (treatment effect).

C: You will be asked to consider your standard deviation of the PREE pain score at 12 months after a total elbow replacement and Distal Humerus Hemiarthroplasty.

**The PREE score**

The PREE is a validated 20 item pain and function score used to measure outcomes of elbow interventions. It is divided into 5 questions on pain, and 15 questions on function. The two are reported individually on a scale from 0 to 50, and the two can be combined to give a total score on a scale from 0 to 100. For this elicitation exercise you are only asked to consider the difference in the median pain scores. In the PREE the five items below are scored on a numerical rating scale from 0 to 10 and the scores for each are summed to give a total pain score out of 50.

1. Rate your pain when you are at rest
2. Rate your pain when doing a task with repeated arm movement
3. Rate your pain when lifting a heavy object
4. Rate your pain at its worst
5. How often do you have pain? (0 = never, 10 = always)

**Background**

The number of fractures of the distal humerus is increasing due to the ageing population.(1) Whilst fixation of the fracture is desirable, in the older population there is a greater risk of osteoporosis and more complex fractures that mean fixation is not possible, and a randomised controlled trial has indicated that joint replacement (arthroplasty) may be a better option for these patients.(2) The increasing demand is reflected by a 3-fold increase in the number of elbow arthroplasty procedures for acute trauma recorded by the National Joint Registry (NJR) over 10 years.(3) There are two available options for prosthetic replacement for trauma, total elbow arthroplasty (TEA) where both sides of the elbow joint are replaced and distal humerus hemiarthroplasty (DHH) where only the broken distal humerus part is replaced. There are some situations where there is a clear indication for TEA, including fractures on both sides of the joint (distal humerus and coronoid fracture) and pre-existing symptomatic arthritis of the elbow. There are also areas where it might be considered reasonable to favour TEA over DHH such as in a patient with known polyarticular inflammatory joint disease. However, for the majority of patients with an acute unreconstructable distal humerus fracture the choice between TEA and DHH is unclear. Currently approximately half of the acute trauma patients recorded in the NJR are having a TEA and half a DHH, suggesting equipoise amongst the orthopaedic community.(3)

Current evidence suggests that there are no significant differences in the outcome for patients from TEA or DHH for acute trauma as measured by functional scores and range of movement, however the quality if the evidence is low.(4)(5) Data from the NJR indicates that there may be differences in revision rate for TEA and DHH but given the small number of cases available this difference is uncertain.(3) A recent Delphi study has determined core outcome domains for elbow trauma and the Patient and Public Involvement group were clear that, of the chosen domains, pain was the most important outcome from their perspective.

**Evidence regarding pain outcomes after elbow arthroplasty for fracture.**

A systematic review of the literature from 2000 to 2023 undertaken for the purposes of this elicitation to explore the pain outcomes of TEA and DHH for acute fractures identified 1 published randomised controlled trial which reported aggregate Likert pain scores for TEA and DHH as measured using the Mayo elbow performance score, the authors were contacted to try to obtain individual pain data but the data is not available.(6) A further trial protocol was identified and the authors contacted to obtain any available data. The authors kindly supplied their data which reported pain as measured by the Oxford Elbow Score and Disabilities of the Arm Shoulder and Hand score but the numbers were below the inclusion criteria for the systematic review (7 patients).(unpublished data) The remaining evidence consisted of case series. There were 17 case series reporting pain outcomes for TEA; 4 reported pain using numerical rating scales (NRSpain) and 14 used a Likert scale for pain. In two of the 5 studies using NRSpain the mean or median was provided but with no estimate of variance so this was imputed from the minimum-maximum values as described by Hozo et al.(7) There were 6 case series reporting pain outcomes for DHH; 2 using NRSpain and 4 using Likert pain scales. The included studies are shown below.

Table 1: Included studies TEA. (8) (9)(10)(11)(12)(13)(14)(15)(16)(17)(18)(19)(6)(20)

Table 2: Included studies DHH.(21)(22)(23)(24)(25)(6)

No direct comparison can be drawn between the data for TEA and DHH due to the risk of bias of included studies but a meta-analysis of the average pain outcome using a NRSpain from 0-10 is shown below with an estimate of the mean for TEA and DHH.


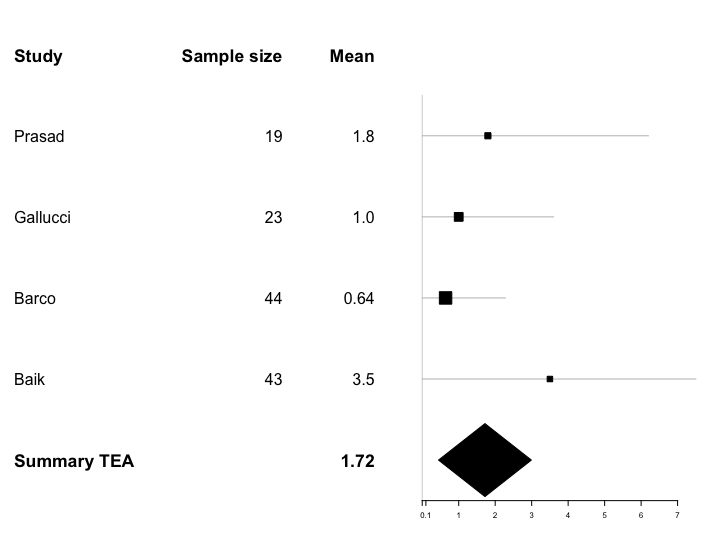


| Studies | 4 | Mean | 1.7193 |
| --- | --- | --- | --- |
| Observations | 129 | LL | 0.4464 |
|  |  | UL | 2.9921 |
|  |  | I2(%) | 95.9 |
|  |  | Q | 72.62 |
|  |  | DF | 3 |
|  |  | P | <0.0001 |

Figure 1. Results of meta-analysis of NRS pain scores for TEA


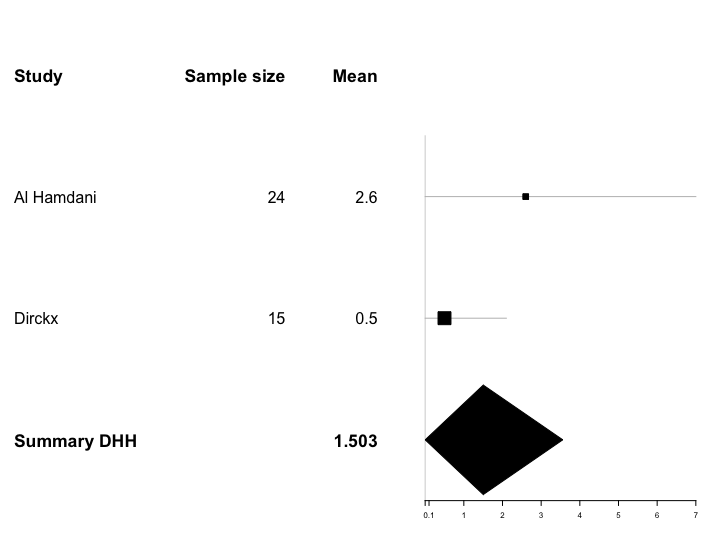


| Studies | 2 | Mean Pain | 1.503 |
| --- | --- | --- | --- |
| Observations | 39 | LL | 0.001 |
|  |  | UL | 3.5589 |
|  |  | I2(%) | 93.6 |
|  |  | Q | 15.6 |
|  |  | DF | 1 |
|  |  | P | <0.0001 |

Figure 2. Results of meta-analysis of NRS pain scores for DHH

A sensitivity analysis was undertaken including only those studies in which the mean and standard deviation was reported. This did not change to results for DHH but for TEA the summary NRS Pain value was reduced to 1.00.

A meta-analysis of results from the published Likert scales for pain are reported below with the probabilities and 95% confidence intervals for outcomes (no pain, mild pain, moderate pain, severe pain). Reported differences in proportions for TEA and DHH may be due to heterogeneity of inclusion criteria and the risk of bias of evidence.

Fig 3. Summary results Likert pain scale TEA.
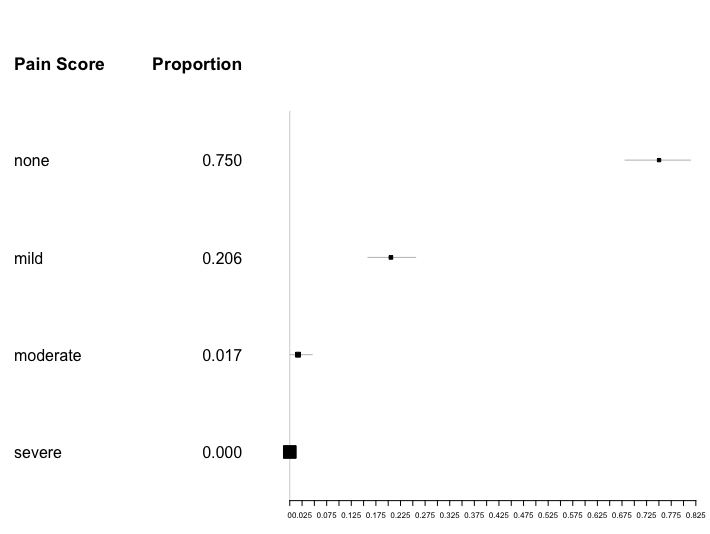


| Studies | 15 |  | None | Mild | Moderate | Severe |
| --- | --- | --- | --- | --- | --- | --- |
| Observations | 306 | Events | 227 | 66 | 11 | 2 |
|  |  | Proportion | 0.7503 | 0.2055 | 0.0167 | 0.0001 |
|  |  | LL | 0.6813 | 0.159 | 0.0007 | 0.0001 |
|  |  | UL | 0.8142 | 0.2558 | 0.0455 | 0.0105 |
|  |  | I2(%) | 41.5 | 0 | 26.8 | 0 |
|  |  | Q | 23.93 | 13.84 | 19.14 | 5.19 |
|  |  | DF | 14 | 14 | 14 | 14 |
|  |  | P | 0.0467 | 0.4615 | 0.1598 | 0.983 |

Fig 4. Summary results Likert pain scale DHH.
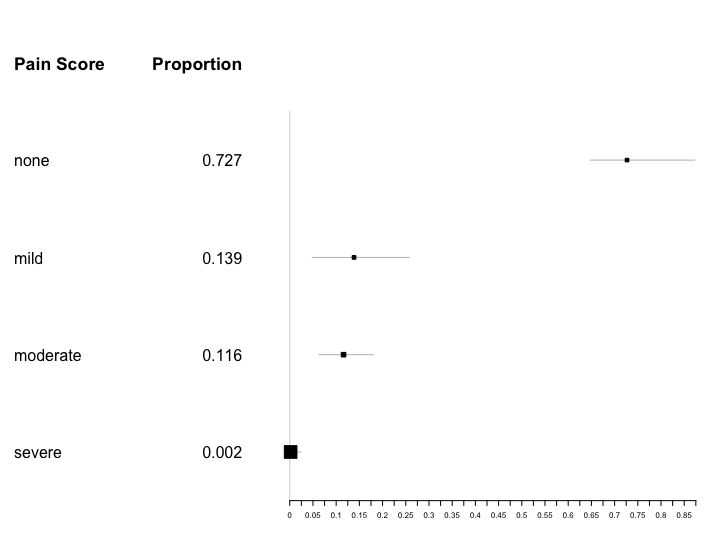


| Studies | 6 |  | None | Mild | Moderate | Severe |
| --- | --- | --- | --- | --- | --- | --- |
| Observations | 149 | Events | 106 | 23 | 19 | 1 |
|  |  | Proportion | 0.7267 | 0.1385 | 0.1159 | 0.0019 |
|  |  | LL | 0.6476 | 0.0486 | 0.0625 | 0 |
|  |  | UL | 0.8726 | 0.2581 | 0.1806 | 0.0245 |
|  |  | I2(%) | 76.5 | 66.4 | 25.2 | 0 |
|  |  | Q | 21.32 | 14.86 | 6.69 | 0.83 |
|  |  | DF | 2 | 5 | 5 | 5 |
|  |  | P | 0.0007 | 0.011 | 0.245 | 0.9748 |

**Pain instruments**

The choice of instruments to measure pain after total elbow arthroplasty is limited. Many instruments combine pain assessment with function assessment (Oxford elbow score, DASH score, Liverpool elbow score) and the items are rarely reported separately by investigators. Pain scales such as the visual analogue scale, numerical rating scale or Likert scale have been validated as tools for measuring pain but we are not aware of any investigations of their psychometric properties in elbow arthroplasty studies. The Patient Rated Elbow Evaluation (PREE) and American Shoulder and Elbow Society (ASES) scores measure and report pain independently and have been validated in general elbow conditions and in total elbow arthroplasty studies.(26)(27) The PREE is less resource intensive as it does not require physician assessment.

Angst have reported that in patients undergoing total elbow replacement the mean PREE improved from a score of 30/50 (s.d. 9.2) pre-operation to 15/50 (s.d. 13.7) at 6 months after surgery. 54% of the 79 patients included had a diagnosis of rheumatoid arthritis.(28) Acute fracture patients were excluded from the analysis.

In a separate study Angst has measured pain six months after TER using PREE pain and a Likert pain scale from the SF-36.(27) The mean PREE pain score was 14/50 (s.d. 13.3) and the mean SF-36 pain score was 60 which equates to “mild pain” on the 6-item SF-36 Likert scale.

**References**

1. Palvanen M, Kannus P, Niemi S, Parkkari J. Secular trends in distal humeral fractures of elderly women. Bone. 2010 May;46(5):1355–8.

2. McKee MD, Veillette CJH, Hall JA, Schemitsch EH, Wild LM, McCormack R, et al. A multicenter, prospective, randomized, controlled trial of open reduction—internal fixation versus total elbow arthroplasty for displaced intra-articular distal humeral fractures in elderly patients. J Shoulder Elbow Surg. 2009 Jan;18(1):3–12.

3. 19th Annual Report of the National Joint Registry [Internet]. NJR; 2022. Available from: https://reports.njrcentre.org.uk/Portals/0/PDFdownloads/NJR%2019th%20Annual%20Report%202022.pdf

4. Stoddart MT, Panagopoulos GN, Craig RS, Falworth M, Butt D, Rudge W, et al. A systematic review of the treatment of distal humerus fractures in older adults: A comparison of surgical and non-surgical options. Shoulder Elb. 2022 May 9;175857322210998.

5. Burden EG, Batten T, Smith C, Evans JP. Hemiarthroplasty or total elbow arthroplasty for unreconstructable distal humeral fractures in patients aged over 65 years: a systematic review and meta-analysis of patient outcomes and complications. Bone Jt J. 2022 May 1;104-B(5):559–66.

6. Jonsson EÖ, Ekholm C, Hallgren HB, Nestorson J, Etzner M, Adolfsson L. Elbow hemiarthroplasty and total elbow arthroplasty provided a similar functional outcome for unreconstructable distal humeral fractures in patients aged 60 years or older: a multicenter randomized controlled trial. J Shoulder Elbow Surg. 2023 Sep;S105827462300705X.

7. Hozo SP, Djulbegovic B, Hozo I. Estimating the mean and variance from the median, range, and the size of a sample. BMC Med Res Methodol. 2005 Dec;5(1):13.

8. Sørensen BW. Primary total elbow arthroplasty in complex fractures of the distal humerus. World J Orthop. 2014;5(3):368.

9. Prasad N, Dent C. Outcome of total elbow replacement for distal humeral fractures in the elderly: A COMPARISON OF PRIMARY SURGERY AND SURGERY AFTER FAILED INTERNAL FIXATION OR CONSERVATIVE TREATMENT. J Bone Joint Surg Br. 2008 Mar;90-B(3):343–8.

10. Prasad N, Ali A, Stanley D. Total elbow arthroplasty for non-rheumatoid patients with a fracture of the distal humerus: a minimum ten-year follow-up. Bone Jt J. 2016 Mar;98-B(3):381–6.

11. Pogliacomi F, Schiavi P, Defilippo M, Corradi M, Vaienti E, Ceccarelli F, et al. Total elbow arthroplasty following complex fractures of the distal humerus: results in patients over 65 years of age. Acta Bio-Medica Atenei Parm. 2016 Sep 13;87(2):148–55.

12. Kamineni S, Morrey BF. Distal Humeral Fractures Treated with Noncustom Total Elbow Replacement: J Bone Jt Surg. 2004 May;86(5):940–7.

13. Garcia JA, Mykula R, Stanley D. Complex fractures of the distal humerus in the elderly. The role of total elbow replacement as primary treatment. J Bone Joint Surg Br. 2002 Aug;84(6):812–6.

14. Gambirasio R, Riand N, Stern R, Hoffmeyer P. Total elbow replacement for complex fractures of the distal humerus. An option for the elderly patient. J Bone Joint Surg Br. 2001 Sep;83(7):974–8.

15. Gallucci GL, Larrondo Calderón W, Boretto JG, Castellaro Lantermo JA, Terán J, De Carli P. Total elbow arthroplasty for the treatment of distal humeral fractures. Rev Esp Cir Ortopédica Traumatol Engl Ed. 2016 May;60(3):167–74.

16. Frankle MA, Herscovici, D, DiPasquale TG, Vasey MB, Sanders RW. A Comparison of Open Reduction and Internal Fixation and Primary Total Elbow Arthroplasty in the Treatment of Intraarticular Distal Humerus Fractures in Women Older Than Age 65: J Orthop Trauma. 2003 Aug;17(7):473–80.

17. Barco R, Streubel PN, Morrey BF, Sanchez-Sotelo J. Total Elbow Arthroplasty for Distal Humeral Fractures: A Ten-Year-Minimum Follow-up Study. J Bone Jt Surg. 2017 Sep 20;99(18):1524–31.

18. Antuña SA, Laakso RB, Barrera JL, Espiga X, Ferreres A. Linked total elbow arthroplasty as treatment of distal humerus fractures. Acta Orthop Belg. 2012 Aug;78(4):465–72.

19. Ali A, Shahane S, Stanley D. Total elbow arthroplasty for distal humeral fractures: indications, surgical approach, technical tips, and outcome. J Shoulder Elbow Surg. 2010 Mar;19(2 Suppl):53–8.

20. Celli A, Paroni C, Bonucci P, Celli L. Total elbow arthroplasty for acute distal humeral fractures with humeral condyle resection or retention: a long-term follow-up study. JSES Int. 2021 Jul;5(4):797–803.

21. Al-Hamdani A, Rasmussen JV, Sørensen AKB, Ovesen J, Holtz K, Brorson S, et al. Good outcome after elbow hemiarthroplasty in active patients with an acute intra-articular distal humeral fracture. J Shoulder Elbow Surg. 2019 May;28(5):925–30.

22. Nestorson J, Ekholm C, Etzner M, Adolfsson L. Hemiarthroplasty for irreparable distal humeral fractures: medium-term follow-up of 42 patients. Bone Jt J. 2015 Oct;97-B(10):1377–84.

23. Rotini R, Ricciarelli M, Guerra E, Marinelli A, Celli A. Elbow hemiarthroplasty in distal humeral fractures: Indication, surgical technique and results. Injury. 2020 Nov;S0020138320309463.

24. Celli A, Ricciarelli M, Guerra E, Bonucci P, Ritali A, Cavallo M, et al. Elbow hemiarthroplasty for acute distal humeral fractures and their sequelae: medium- and long-term follow-up of 41 cases. J Shoulder Elbow Surg. 2022 May;31(5):1015–25.

25. Dirckx M, Tathgar A, Bellringer S, Phadnis J. Hemiarthroplasty versus open reduction internal fixation for intra-articular distal humerus fractures in older patients. Shoulder Elb. 2023 Feb;15(1):83–92.

26. Vincent JI, MacDermid JC, King GJW, Grewal R. The Patient-Rated Elbow Evaluation and the American Shoulder and Elbow Surgeons—Elbow form capture aspects of functioning that are important to patients with elbow injuries. J Hand Ther. 2021 Jul;34(3):415–22.

27. Angst F, John M, Pap G, Mannion AF, Herren DB, Flury M, et al. Comprehensive assessment of clinical outcome and quality of life after total elbow arthroplasty. Arthritis Care Res. 2005 Feb 15;53(1):73–82.

28. Angst F, Goldhahn J, Drerup S, Kolling C, Aeschlimann A, Simmen BR, et al. Responsiveness of five outcome measurement instruments in total elbow arthroplasty. Arthritis Care Res. 2012 Nov;64(11):1749–55.
