# Supplementary material for: Pain score distribution: an expert elicitation study for distal humerus hemiarthroplasty and total elbow arthroplasty using a validated numerical patient rated outcome-measure in trauma
Source: JSES Int. 2026 Jan 27;10(3):101640. doi: 10.1016/j.jseint.2026.101640 (PMC13054023; doi:10.1016/j.jseint.2026.101640)
Supplement: Supplementary Appendix S3 [file mmc3.docx]

Appendix 3: Expert enquiry form and responses.

| **Name and Title** | **Background and Expertise** | **Declaration of Interests** | **Additional Evidence** | **Clarifications and Corrections** |
| --- | --- | --- | --- | --- |
| MANSAT Pierre, Prof. | Master in Biomechanics 1992; Fellowship Mayo Clinic - Adult Reconstruction Upper Limb (Shoulder-Elbow) 1996/1997; PhD Thesis on Shoulder arthroplasty using FEA 2002; Professor of Orthopedic and Traumatology, University Hospital Toulouse FRANCE 2004; Main activities: Shoulder, Elbow and Hand Surgeries; 180 per-reviewed articles indexed in Pubmed; Chief-editor JSES International (Elsevier) | Chief-editor JSES International (Elsevier); Payed consultant: Medartis, Stryker, Zimmer-Biomet | None | None |
| Lars Adolfsson, Professor, MD | Specialist in orthopedic and hand surgery. 35 years experience. Over 100 peer reviewed publications on upper extremity trauma and surgery. Several on elbow arthroplasty | none | nothing very important missing | none |
| Toni Luokkala, MD PhD, Consultant Elbow and Hand Surgeon | Current employer: Wellbeing services county of Central Finland, Hospital Nova, Jyväskylä, Finland. Background: 12 years as a consultant elbow and hand surgeon including upper limb fellowship position in Wrightington hospital 2017. | Consultant, National patient insurance center, Finland | - | - |
| Joideep Phadnis | Elbow specialist | Consultant for stryker (arthroplasty implants) | none additional | none |
| Jonathan Evans | I am a Consultant Shoulder and Elbow surgeon at the Princess Elizabeth Orthopaedic Centre, Royal Devon University Hospital, Exeter. I also hold a position as a Senior Clinical Lecturer in Orthopaedics at the University of Exeter. As a clinical academic I split my time 50/50 between clinical work and research activity. Over the last few years I have published systematic reviews and meta-analysis on the outcome of total elbow replacements and distal humeral hemiarthroplasties in the context of trauma and have conducted a feasibility randomised controlled trial assessing these two interventions in unreconstructable distal humeral fractures. In 2017 I completed my doctorate assessing outcomes in lateral elbow tendinopathy, a major component of which involved the assessment of the psychometric properties of elbow specific Patient Reported Outcome Measures (PROMs). I have maintained an interest in PROMs and have published on core outcome sets in elbow pathology and the use of advanced psychometric techniques including item response theory and computerised adaptive tests. | I have no relevant disclosures | I have read the dossier and there is no relevant further published evidence I would offer. I have discussed some pre-published data with Prof Watts from our feasibility RCT. | No further clarifications and corrections. |
| Andrew Wright. Consultant Elbow & Shoulder Surgeon, Wrightington Hospital. | Consultant Elbow Surgeon at Wrightington Hospital UK since 2020. My practice is predominantly elbow orientated with tertiary referrals from regional colleagues. I have collaborated in a multidisciplinary working group to produce national guidelines for lateral elbow tendinopathy. | No interests to declare | No additional | N/A |
| Alexander Van Tongel MD PhD | Shoulder - Elbow surgeon University Hospital Ghent | To evaluate the average pain score and to help to lower the pain score with new techniques | - | - |
